# Using DTI to assess white matter microstructure in cerebral small vessel disease (SVD) in multicentre studies

**DOI:** 10.1042/CS20170146

**Published:** 2017-06-07

**Authors:** Iain D. Croall, Valerie Lohner, Barry Moynihan, Usman Khan, Ahamad Hassan, John T. O’Brien, Robin G. Morris, Daniel J. Tozer, Victoria C. Cambridge, Kirsty Harkness, David J. Werring, Andrew M. Blamire, Gary A. Ford, Thomas R. Barrick, Hugh S. Markus

**Affiliations:** 1 Department of Clinical Neuroscience, Stroke Research Group, University of Cambridge, Cambridge, U.K.; 2 Neurosciences Research Centre, St. George's, University of London, London, U.K.; 3 Stroke and Dementia Research Centre, St George’s, University of London, London, U.K.; 4 Department of Neurology, Leeds General Infirmary, Leeds Teaching Hospitals NHS Trust, Leeds, U.K.; 5 Department of Psychiatry, University of Cambridge, Cambridge, U.K.; 6 Department of Psychology, Kings' College London, Institute of Psychiatry, Psychology and Neuroscience, London, U.K.; 7 Department of Neurology, Royal Hallamshire Hospital, Sheffield, U.K.; 8 Department of Brain Repair and Rehabilitation, Stroke Research Centre, UCL Institute of Neurology, London, U.K.; 9 Magnetic Resonance Centre, Institute of Cellular Medicine, Newcastle University, Newcastle, U.K.; 10 Medical Sciences Division, University of Oxford, Oxford, U.K., & Oxford University Hospitals NHS Foundation Trust; 11 Molecular and Clinical Science Research Institute, St George’s, University of London, London, U.K.

**Keywords:** biomarkers, cerebral small vessel disease, clinical trials, cognition, diffusion tensor imaging

## Abstract

Diffusion tensor imaging (DTI) metrics such as fractional anisotropy (FA) and mean diffusivity (MD) have been proposed as clinical trial markers of cerebral small vessel disease (SVD) due to their associations with outcomes such as cognition. However, studies investigating this have been predominantly single-centre. As clinical trials are likely to be multisite, further studies are required to determine whether associations with cognition of similar strengths can be detected in a multicentre setting. One hundred and nine patients (mean age =68 years) with symptomatic lacunar infarction and confluent white matter hyperintensities (WMH) on MRI was recruited across six sites as part of the PRESERVE DTI substudy. After handling missing data, 3T-MRI scanning was available from five sites on five scanner models (Siemens and Philips), alongside neuropsychological and quality of life (QoL) assessments. FA median and MD peak height were extracted from DTI histogram analysis. Multiple linear regressions were performed, including normalized brain volume, WMH lesion load, and n° lacunes as covariates, to investigate the association of FA and MD with cognition and QoL. DTI metrics from all white matter were significantly associated with global cognition (standardized β =0.268), mental flexibility (β =0.306), verbal fluency (β =0.376), and Montreal Cognitive Assessment (MoCA) (β =0.273). The magnitudes of these associations were comparable with those previously reported from single-centre studies found in a systematic literature review. In this multicentre study, we confirmed associations between DTI parameters and cognition, which were similar in strength to those found in previous single-centre studies. The present study supports the use of DTI metrics as biomarkers of disease progression in multicentre studies.

## Introduction

Cerebral small vessel disease (SVD) causes a quarter of all ischaemic strokes, is the most common pathology underlying vascular cognitive impairment and dementia [1] and contributes to the severity of Alzheimer’s disease [2]. SVD affects the small vessels of the brain and results in a number of characteristic radiological appearances best seen on MRI, including lacunar infarcts, T2-white matter hyperintensities (WMH), cerebral microbleeds, and brain atrophy [3,4]. In terms of symptoms, cognitive impairment may be the most debilitating [5], with SVD characteristically associated with early deficits in executive function and processing speed, while episodic memory is relatively spared [1,2,6–9].

Despite the public health importance of SVD, there are few specific treatments [10]. Furthermore, evaluating treatments represent a major challenge due to the variable rate of cognitive decline, which can be slow in many patients, but occurs rapidly with progression to dementia in a subset. While cognitive testing plays a central role in identifying the presence of cognitive impairment, it has proved to be relatively insensitive to longitudinal change [11]. This has led to the suggestion that MRI might represent a useful surrogate marker to monitor disease progression and evaluate the efficacy of therapeutic interventions in smaller number of patients prior to larger phase III trials [3,12].

Diffusion tensor imaging (DTI) has been shown to be particularly sensitive to white matter damage in SVD. Abnormalities have been shown not only within T2-WMH but also in apparently ‘normal appearing white matter’ [13], and these changes correlate better with cognition than WMH lesion volume [8]. In single-centre studies, change in DTI could be detected in SVD patients over follow-up periods of 1–3 years [14,15]. This has led to the suggestion that DTI might provide a useful surrogate marker and power calculations for phase II trials based on the rate of DTI change seen in these papers, which show that its use may allow evaluation of therapeutic interventions with much smaller sample sizes than if cognitive function was used as an outcome measure [11]. However, studies conducted to date have been single-centre [12,15,16]. Most therapeutic trials are likely to be multicentre and involve acquisition of DTI across different sites. As image acquisition will be on different scanners, this may present challenges [17]. It is important to assess whether DTI is feasible in a clinical trial setting, and whether similar associations between MRI parameters and clinical and cognitive variables can be detected in the multicentre setting. One way of assessing this is to determine whether the strength of association between DTI and cognition in multicentre studies is similar to that previously reported in single-centre studies.

To evaluate this, we determined the association between DTI parameters and cognition in the baseline data of a multicentre trial.

## Methods

### PRESERVE study

The PRESERVE study (‘How intensively should we treat blood PRESsure in established cERebral small VEssel disease?’) is a multicentre randomized controlled trial comparing a strategy of intensive compared with standard treatment of blood pressure on cognitive function over a 2-year follow-up period. Nested within the overall study is a DTI substudy in which patients additionally undergo multimodal MRI including DTI at baseline and at the end of the 2-year follow-up period. The baseline data from these individuals are presented in the present paper.

### Study population

Inclusion criteria were: a clinical lacunar stroke with an anatomically corresponding lacunar infarct on MRI, in addition to confluent WMH graded as ≥2 on the Fazekas scale [18]. Patients were at least 40 years old with hypertension defined as either a systolic blood pressure >140 mmHg, or a systolic blood pressure between 125–140 mmHg while on antihypertensive treatment. Exclusion criteria were: a known single gene disorder causing SVD (e.g. CADASIL), symptomatic carotid stenosis or vertebral stenosis >50%, cortical infarction >2 cm diameter, diagnosis of dementia, life expectancy of less than 2 years, symptomatic postural hypotension, women with childbearing potential and any inability to fulfil study data collection. All patients gave informed written consents. The study was approved by the Harrow National Research Ethics Service committee (“REC” number: 11/LO/0458) and is registered with the U.K. Clinical Research Network (CRN number: 10962).

One hundred and nine patients from six sites consented to participate in the PRESERVE DTI substudy. The site sample sizes are as follows: site 1 (*n*=48), site 2 (*n*=29), site 3 (*n*=14), site 4 (*n*=11), site 5 (*n*=6), site 6 (*n*=1). Participants underwent baseline testing at least 3 months post-stroke.

### Clinical assessments

A stroke physician or vascular neurologist examined all the participants. Cerebrovascular risk factors including a history of previous stroke, hypercholesterolaemia, diabetes, smoking (current and history), angina, myocardial infarction, coronary artery bypass grafts (CABGs) or coronary angioplasty were recorded.

### Neuropsychological assessment

#### Cognitive testing

Assessment was performed by a neuropsychologist and occurred on the same day as MRI scanning or as close to the scan as possible. A cognitive test battery was used which included tests sensitive to the characteristic impairments in processing speed and executive function associated with SVD [2], with additional testing of memory. This included for Processing speed the Digit Symbol Coding test (DSC) [19], and for executive functioning the Trail Making Test (TMT, [20]) to measure mental flexibility and a phonemic verbal fluency task ("FAS") [21] and a semantic verbal fluency task (animals) [22] to measure verbal generativity.Memory was measured using the Rey Auditory Verbal Learning Test (RAVLT, [23]). Premorbid IQ was estimated using the restandardized National Adult Reading Test (NART-R, [24]) and additional screening for cognitive impairment was conducted using the Montreal Cognitive Assessment (MoCA, [25]).

In addition, following assessments of disability and quality of life (QoL) were performed; the stroke-specific QoL assessment (SSQoL) [26] and the EuroQoL [27].

Performance across neuropsychological tests was made comparable by transforming raw scores into z-scores using the best available age-scaled normative data (DSC; [19], TMT; [28], letter fluency; [21], animal fluency; [22], RAVLT; [28]). Tasks were grouped into four key domains (**Processing speed**: Wechsler Adult Intelligence Scale coding total correct, TMT-A time to complete, **Mental flexibility**: TMT-B time to complete, **Verbal fluency**: total correct for ‘FAS’ letter fluency and animal fluency and **Verbal memory**: RAVLT ‘immediate’ and ‘delayed’ recall). Individual task z-scores were averaged across these groupings to create overall domain scores, while all domain scores were averaged to create a **Global cognition** domain. SSQoL (total score), EuroQoL (‘healthstate’ rating) and the MoCA (total score) were analysed individually using raw scores.

Where data were missing due to a subject being unable to complete a task, the lowest available z-score was given; this applied to 15 individual tasks, across 13 participants (11.9% of the sample size). If data were missing for any other reasons then the domain scores were calculated without that task; this applied to three participants (2.8% of the sample size).

### MRI acquisition

The aim was to test a study design for which MRI data were acquired using clinical scanners in different sites from different manufacturers. Within the six centres, eight 3-Tesla MR scanners were used (three Philips Achieva TX, one Philips Achieva, one Philips Ingenia, one Siemens Verio, one Siemens Prisma, one Siemens Magnetom Prisma fit). MRI acquisition included 3D T1-weighted (T1W), and DTI, T2*-weighted (T2*W) and Fluid-attenuated Inversion Recovery (FLAIR) scans for each participant. A rigorous quality control was implemented to ensure sequence acquisition parameters were as standardized as possible. T1W scans were acquired at 1-mm^3^ isotropic voxel resolution and TR and TE were optimized to ensure comparable T1 weighting and tissue contrast across sites. DTI scans (2-mm^3^ isotropic voxel resolution) had similar TEs and long TRs to avoid T1 relaxation effects. In addition to the b =0 s mm^−2^ acquisitions, all DTI acquisition included 32 equally spaced, non-collinear diffusion gradient directions (b =1000 s mm^−2^) to ensure identical angular resolution and noise characteristics. T2*W sequences were TE matched and kept a similar TR to ensure comparable weighting. FLAIR sequences had identical inversion times and were also TE matched with long enough TRs to ensure no T1 weighting occurred. Resolution for T2*W and FLAIR sequences varied between sites; Supplementary Table S1 gives an overview of the exact scanner and sequence details per site.

**Table 1:** Baseline characteristics of the study population

Demographic variables	Mean (S.D.)/number (%)
Age, mean (S.D.) years	68.2 (9.07)
Male, *n* (%)	64 (58.7%)
Premorbid IQ	115.8 (8.12)
MoCA <26	54 (49.5%)
Systolic blood pressure (mmHg)	150 (13)
Diastolic blood pressure (mmHg)	85 (12)
Previous stroke, *n* (%)	21 (19.3%)
Hypercholesterolaemia, n (%)	84 (77.1%)
Diabetes, *n* (%)	24 (22.0%)
Current smokers, *n* (%)	16 (14.7%)
Former smokers, *n* (%)	40 (37.7%)
Angina, *n* (%)	7 (6.4%)
Myocardial infarction, CABG or coronary angioplasty, *n* (%)	6 (5.5%)
Peripheral vascular disease, *n* (%)	2 (1.9%)
History of depression,* n* (%)	20 (18.3%)

Other missing data not previously reported; former smoker =3; peripheral vascular disease =1.

### MRI data analysis

In addition to DTI, measures describing WMH, lacunes and brain volume are frequently investigated as potential markers of SVD [8,12,29–31]. In the present study, these were analysed as a comparison with DTI.

#### WMH

WMH were defined as areas of increased signal on FLAIR images (excluding the rims of cavitated lacunes) and segmented by a single trained rater (I.D.C.) using a semi-automated, contouring technique in Jim image analysis software version 7.0_5 (Xinapse Systems Limited, http://www.xinapse.com/j-im-7-software/). Whole brain WMH lesions maps were generated and a WMH lesion load score was calculated as the percentage of WMH lesion volume against whole brain volume. To assess intra- and inter-rater reliability, a test set of ten FLAIR scans (from a previous study in SVD) with varying degrees of WMH was used. In a randomized, blinded setting FLAIR images were each marked twice by I.D.C. and once by a second experienced rater (D.T.). The intraclass correlation coefficient [32] was calculated to assess inter-rater reliability (I.D.C. compared with D.T.) and intrarater reliability providing coefficients of 0.988 and 0.998 respectively.

#### Lacunes

Lacunes were defined as cerebrospinal fluid (CSF)-filled cavities at least 3 mm in diameter. Additional features such as T2-hyperintense rims, shape and location were also considered to differentiate lacunes from similar imaging features such as perivascular spaces. The same single rater (I.D.C.) identified lacunes after training by a consultant neuroradiologist using a combination of T1W, T2*W and FLAIR scans.

#### Brain volume

T1W scans were intensity non-uniformity corrected using ‘N4ITK’ [33] and segmented into grey matter (GM), white matter (WM) and CSF tissue probability maps (TPM) using SPM12b (Statistical Parametric Mapping (SPM), http://www.fil.ion.ucl.ac.uk/spm/ [34]). Brain volume in native space was calculated from the soft segmentation of the GM and WM TPMs.

To obtain brain volume measures sensitive to atrophy, "SIENAX" ( [35], a part of FMRIB Software Library (FSL), https://fsl.fmrib.ox.ac.uk/fsl [36]) was applied to T1W scans giving a scaling factor that describes the variation of brain size relative to the skull size. The native space brain volumes were multiplied by this scaling factor to provide normalized brain volumes (NBVs). To minimize the tissue misclassification of WMH as GM, the (normalized) volume of any GM which occurred within WMH was subtracted from the GM volume and added to the WM volume. Finally, whole NBV was calculated by adding GM and WM NBVs together.

#### DTI histogram analysis

FSL software ("FDT"; FMRIB’s Diffusion Toolbox, http://fsl.fmrib.ox.ac.uk/fsl/fslwiki/FDT) was used for DTI preprocessing. Briefly, DTI scans were eddy current corrected with eddy_correct using the first acquired b =0 s mm^−2^ image as the reference. A binary brain mask in DTI space was calculated for each subject using "BET" on the same b =0 acquisition. Fractional anisotropy (FA) and mean diffusivity (MD) maps were then calculated from these data using "DTIFIT". Voxels with MD values above 0.0026 mm^2^s^−1^ were removed from analyses in case they had been misclassified as CSF voxels by application of a diffusivity threshold. Likewise, spurious voxels with FA >1 were also removed. For each participant, FMRIB Linear Image Registration Tool (FLIRT, [37], using the normalized mutual information cost function in FSL) was used to register the FLAIR to the T1W image and the T1W to the b0 image (the average of all the b =0 s mm^−2^ images in the DTI sequence). These affine transformation matrices were concatenated to create a third FLAIR-to-DTI transformation. TPMs and WMH lesion masks were registered into DTI space using the T1W-to-DTI (trilinear interpolation) and FLAIR-to-DTI (nearest neighbour interpolation) transforms for TPMs and binary WMH lesion masks respectively.

A hard segmentation method was applied to generate maps of tissue classes. This was achieved by voxelwise comparison of the GM, WM and CSF TPMs, with each voxel being assigned to the highest probability tissue class. The WMH lesion masks were then added with these lesion voxels being automatically assigned to WMH. Finally, mask images of normal appearing white matter (NAWM) and all white matter (WM) were generated from the hard segmentation map.

Histogram analysis was performed on FA and MD maps in both NAWM and WM. Normalized histograms with 1000 bins (FA range: 0–1, bin width: 0.001; MD range: 0–4 mm^2^s^−1^ × 10^−3^, bin width: 0.004 mm^2^s^−1^ × 10^−3^) were computed and median, peak height and peak value were extracted from these for both FA and MD. These metrics were chosen as summary measures as FA and MD are non-normally distributed in WM.

#### Statistical analyses

All analyses were performed using IBM SPSS statistics version 23 (IBM Corp. Released 2013. IBM SPSS Statistics for Windows. Armonk, NY: IBM Corp, http://www.ibm.com/analytics/us/en/technology/spss/).

One measure for each MD and FA was chosen for the main study analyses. MD (normalized) peak height and FA median were picked due to previous studies which have shown these to be correlated with cognition [8,14] and sensitive to change in WM microstructure in SVD [11,15].

To compare MRI with cognitive parameters, ‘Simple’ and ‘Complex’ model linear regressions were conducted. This pipeline was structured as a method of selecting the most appropriate MRI measure per type (e.g. one brain tissue volume measurement or MD/FA histogram parameter for DTI) so that contributions of MRI metrics could be assessed together, while avoiding issues of multicollinearity. Thus, in Simple models, the association of NBV, WMH lesion load, lacunes and histogram parameters (from NAWM and WM) were separately investigated against each outcome measure (cognitive domains, QoL and MoCA). As there were multiple NBV and DTI variables, the most significant of each type (or if *P*-value was the same, the one with the largest β-value) per outcome measure, was selected and used in the Complex model. Here, NBV, WMH lesion load, n° lacunes and DTI measures were included together to assess their contributions relative to each other. Separate Complex models were performed for each outcome measure, in WM and NAWM. These models controlled for confounding effects of age, gender, premorbid IQ and were stratified by study site. Residuals were inspected for normality for all regression analyses while variance inflation factors were also calculated for the Complex models to assess multicollinearity.

Further analyses compared DTI and outcome variables between sites and repeated some Complex model analyses using site-specific data. These are detailed in the Supplementary material.

#### Systematic review

To allow comparison of the results with previous single-centre studies, a systematic review of previous literature was conducted on PubMed (https://www.ncbi.nlm.nih.gov/pubmed/) using as search terms ‘cerebral small vessel disease diffusion tensor imaging’, ‘white matter hyperintensities diffusion tensor imaging’ and ‘leukoaraiosis diffusion tensor imaging’ on 16th March, 2017. Criteria for inclusion were: (i) studies of sporadic SVD population (i.e. monogenic causes of SVD such as CADASIL were not included), (ii) studies investigating the relationship between DTI metrics and cognitive performance, (iii) studies investigating the cognitive domains analysed in the current study, (iv) analysis controlling at least one other confounding MRI measure, (v) results involved reporting of standardized β-values or partial correlation coefficients. Where a paper reported multiple associations against the same cognitive outcome, the strongest (i.e. largest β-value) was included. In cases where a study had published multiple papers based on the same participant data, the one which used the most similar metrics to those in the presented study was chosen.

## Results

### Profile of participant variables

#### Missing data

Due to the low sample size (*n*=1), site 6 was excluded from all statistical analyses. An additional six participants were excluded from analysis due to MRI data acquisition problems (two cases from site 1 due to excessive motion artefacts and corrupted data acquisition and four cases from site 4 where not all imaging sequences were acquired and some data were corrupted). Sample size was further reduced by incomplete cognitive data. Verbal fluency data was absent for one participant, Verbal memory and NART in another and (only) NART in third. Sample size was therefore reduced by further three for Verbal fluency comparisons, and by two for all other comparisons. Consequently, complete DTI data were available in 102 participants, while sample size for main statistical analyses was *n*=99 for testing Verbal fluency or *n*=100 for all other outcome measures.

#### Demographics

Demographics, risk factors and clinical features are shown in Table 1.

All entry MRI scans were reviewed centrally by a consultant neurologist. All cases fitted the MRI inclusion criteria except two which had WMH graded on the Fazekas scale of <2. Both were included in analysis as they had multiple lacunes consistent with severe SVD.

#### Cognition

**Figure 1:**
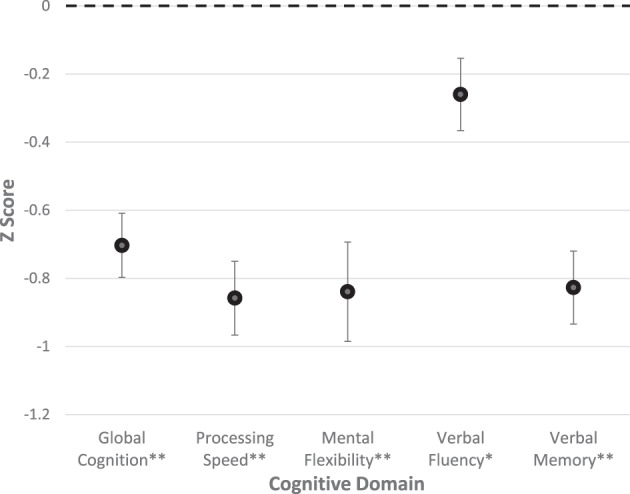
Cognitive profile of the SVD patient group. This figure shows average, age-matched z-scores for cognitive indices. Error bars represent ± 1 S.E.M. Index score significantly different from zero: ** *P≤* 0.001, * *P≤*0.005.

The cognitive profile of the participants is shown in Figure 1. All five cognitive domains were significantly impaired compared with control performance levels (*P*≤0.001 in all cases except for Verbal fluency where *P*≤0.05).

#### MoCA, QoL and MRI results

Mean values for MoCA, SSQoL, EuroQoL and MRI parameters are shown in Table 2. Qualitative comparison of histogram measures between the WM and NAWM tissue classes showed that the inclusion of WMH in the WM lowered the (normalized) peak height of FA and MD, increased the peak value and median of MD, and decreased the peak value and median of FA.

**Table 2:** Mean scores for key individual variables using all available data

Variables	Mean (S.D.), range
**Cognitive/QoL variables**	
MoCA	24.9 (3.5), 11–30
SSQoL	190.6 (32.8), 93–244
EuroQoL	69.3 (19.1), 0–100
**MRI variables**	
NBV (whole brain, ml)	1355.84 (107.70)
GM normalized volume (ml)	714.49 (73.48)
WM normalized volume (ml)	641.35 (70.39)
WMH volume (ml)	34.74 (22.27)
WMH lesion load (% brain)	3.41 (2.22)
Lacunes (number)	4.41 (4.73)
FA height × 10^−3^ (NAWM/WM)	3.27 (0.26)/3.24 (0.25)
MD height × 10^−2^ (NAWM/WM)	1.42 (0.21)/1.33 (0.23)
FA value (NAWM/WM)	0.320 (0.042)/0.311 (0.047)
MD value mm^2^s^−1^ × 10^−3^ (NAWM/WM)	0.761 (0.040)/0.762 (0.040)
FA median (NAWM/WM)	0.342 (0.026)/0.335 (0.028)
MD median mm^2^s^−1^ × 10^−3^ (NAWM/WM)	0.774 (0.039)/0.787 (0.044)

### Relationship between MR variables and cognition

#### Simple model analyses

Full findings are shown in Table 3. FA median and MD peak height (in WM and NAWM) were significantly associated with all outcome measures, except for NAWM MD peak height with Processing Speed, both NAWM measures with SSQoL and all DTI measures with Verbal memory. Median FA held stronger associations than MD peak height in all cases except for EuroQoL in (all) WM. The directions of these relationships demonstrate that higher median FA and MD peak height were associated with better cognition or QoL in both tissue classes. There were no marked differences between the patterns or strengths of associations for DTI measures taken from within NAWM or the whole of the WM. Whole NBV held stronger associations than GM or WM NBV in all cases except EuroQoL, where WM was strongest.

**Table 3:** Results from linear regression ‘Simple model’ analyses

MR variables		Global cog.	Proc. speed	Mental flex.	Verbal fluency	Verbal memory	MoCA	SSQoL	EuroQoL
Volume measures	Whole NBV	**0.330 (0*.003*)**	**0.361 (*0.002*)**	**0.286 (*0.016*)**	0.171 (*0.153*)	**0.245 (*0.041*)**	**0.421 (≤*0.001*)**	0**.273 (*0.036*)**	0.200 (*0.115*)
	Grey NBV	0.167 (*0.109*)	0.109 (*0.321*)	0.111 (*0.312*)	0.147 (*0.177*)	0.177 (*0.109*)	**0.339 (*0.001*)**	0.084 (*0.485*)	–0.016 (*0.888*)
	White NBV	**0.199 (*0.036*)**	**0.283 (*0.004*)**	**0.202 (*0.043*)**	0.049 (*0.627*)	0.101 (*0.320*)	0.142 (*0.150*)	0.213 (*0.051*)	**0.225 (*0.034*)**
WMH measure	Lesion load	**–0.288 (*0.001*)**	**–0.312 (*0.001*)**	**–0.245 (*0.009*)**	**–0.240 (*0.011*)**	–0.132 (0. *170* )	**–0.196 *0.035*)**	**–0.249 (*0.015*)**	**–0.248 (*0.013*)**
Lacune measure	N° lacunes	**–0.357 (≤*0.001*)**	**–0.389 (≤*0.001*)**	**–0.268 (*0.006*)**	**–0.286 (*0.003*)**	**–0.233 (*0.018*)**	**–0.333 (≤*0.001*)**	**–0.323 (*0.002*)**	–0.195 (*0*. *062* )
NAWM DTI measures	FA median	**0.352 (≤*0.001*)**	**0.247 (*0.009*)**	**0.338 (≤*0.001*)**	**0.374 (≤*0.001*)**	0.167 (*0.081*)	**0.332 (≤0.*001*)**	0.196 (*0.058*)	**0.253 (*0.011*)**
	MD peak height	**0.267 (*0.005*)**	0.186 (*0.063*)	**0.241 (*0.016*)**	**0.275 (*0.006*)**	0.160 (*0.115*)	**0.262 (*0.007*)**	0.170 (*0.121*)	**0.244 *(0.021*)**
WM DTI measures	FA Median	**0.371 (≤*0.001*)**	**0.282 (*0.002*)**	**0.354 (≤*0.001*)**	**0.375 (≤*0.001*)**	0.174 (*0.067*)	**0.329 (≤*0.001*)**	**0.213 (*0.037*)**	**0.267 (*0.007*)**
	MD peak height	**0.303 (*0.001*)**	**0.247 (*0.011*)**	**0.273 (*0.005*)**	**0.293 (*0.003*)**	0.162 (*0.101*)	**0.262 (*0.006*)**	**0.218 (*0.042*)**	**0.291 (*0.005*)**

All numbers are standardized β-values (*P*-values). Significant relationships are shown in bold while the most strongly associated MR variable per outcome per category is underlined.

#### Complex model analyses

‘Complex models’ were performed to determine which MRI variables were independently associated with the outcome measures and results are shown in Table 4. The variance inflation factors of all models were smaller than three and deemed acceptable. Median FA was significantly associated with Global cognition, Mental flexibility, Verbal fluency and MoCA in both the tissue classes. No other comparisons with DTI metrics reached significance. Considering the significant associations, the effective sizes of the WM comparisons (as indicated by the β-value) were always descriptively greater than the NAWM counterpart.

**Table 4:** Results from linear regression ‘Complex model’ analyses

Tissue class model	MR variable	Global cog.	Proc. speed	Mental flex.	Verbal fluency	Verbal mem.	MOCA	SSQoL	EuroQoL
NAWM	Whole NBV	0.134 (*0.227*)	0.197 (*0.098*)	0.112 (*0.363*)	–0.041 (*0.735*)	0.163 (*0.213*)	**0.284 (*0.014*)**	0.137 (*0.323*)	–
		[–0.085 : 0.353]	[–0.037 : 0.432]	[–0.131 : 0.356]	[–0.280 : 0.198]	[–0.095 : 0.422]	**[0.058 : 0.509]**	[–0.137 : 0.410]	
	WM NBV	–	–	–	–	–	–	–	**0.230 (*0.030*)**
									**[0.023 : 0.437]**
	WMH lesion load	–0.029 (*0.775*)	–0.126 (*0.246*)	-0.009 (*0.938*)	0.006 (*0.954*)	0.033 (*0.784*)	0.116 (*0.270*)	–0.100 (*0.428*)	–0.144 (*0.315*)
		[–0.230 : 0.172]	[–0.341 : 0.089]	[–0.232 : 0.215]	[–0.212 : 0.225]	[–0.204 : 0.270]	[–0.091 : 0.323]	[–0.351 : 0.150]	[–0.354 : 0.125]
	N° lacunes	**–0.251 (*0.006*)**	**–0.287 (*0.004*)**	–0.166 (*0.099*)	–0.192 (*0.057*)	–0.186 (*0.082*)	**–0.247 (*0.009*)**	**–0.245 (*0.031*)**	–0.047 (*0.672*)
		**[–0.429 : –0.072]**	**[–0.477 : 0.096]**	[–0.365 : 0.032]	[–0.390 : 0.006]	[–0.397 : 0.024]	**[–0.431 : –0.063]**	**[–0.467 : –0.022]**	[–0.266 : 0.172]
	FA median	**0.227 (*0.023*)**	0.038 (*0.717*)	**0.253 (*0.022*)**	**0.333 (*0.002*)**	0.085 (*0.463*)	**0.244 (*0.018*)**	0.032 (*0.796*)	0.196 (*0.099*)
		**[0.032 : 0.421]**	[–0.170 : 0.247]	**[0.037 : 0.470]**	**[0.121 : 0.546]**	[–0.145 : 0.315]	**[0.043 : 0.445]**	[–0.211 : 0.275]	[–0.037 : 0.428]
	MD peak height	–	–	–	–	–	–	–	–
	Model sig. (*P*-value, adj. R^2^)	**<0.001, 0.429**	**<0.001, 0.334**	**<0.001, 0.292**	**<0.001, 0.317**	**0.001, 0.202**	**<0.001, 0.392**	**0.029, 0.108**	**0.004, 0.164**
WM	Whole NBV	0.131 (*0.236*)	0.194 (*0.103*)	0.107 (*0.380*)	–0.041 (*0.730*)	0.162 (*0.215*)	**0.284 (*0.014*)**	0.139 (*0.316*)	–
		[–0.087 : 0.349]	[–0.040 : 0.428]	[–0.135 : 0.350]	[0.280 : 0.197]	[–0.096 : 0.421]	**[0.058 : 0.509]**	[–0.135 : 0.0412]	
	WM NBV	–	–	–	–	–	–	–	**0.225 (*0.033*)**
									**[0.018 : 0.432]**
	WMH lesion load	0.025 (*0.882*)	–0.110 (*0.359*)	0.055 (*0.655*)	0.075 (*0.536*)	0.052 (*0.693*)	0.164 (*0.155*)	–0.100 (*0.474*)	–0.081 (*0.545*)
		[–0.195 : 0.245]	[–0.346 : 0.127]	[–0.189 : 0.299]	[–0.165 : 0.315]	–0.209 : 0.313]	[–0.063 : 0.392]	[–0.376 : 0.176]	[–0.347 : 0.184]
	N°lacunes	**–0.248 (*0.007*)**	**–0.285 (*0.004*)**	–0.163 (*0.104*)	–0.190 (*0.059*)	–0.186 (*0.083*)	**–0.245 (*0.009*)**	**–0.245 (*0.031*)**	–0.047 (*0.669*)
		**[–0.425 : –0.70]**	**[–0.476 : –0.095]**	[–0.360 : 0.034]	[–0.388 : 0.007]	[–0.396 : 0.025]	**[–0.429 : –0.062]**	**[–0.468 : –0.023]**	[–0.267 : 0.172]
	FA median	**0.268 (*0.016*)**	0.058 *(0.621*)	**0.306 (*0.013*)**	**0.376 (*0.002*)**	0.099 (*0.445*)	**0.273 (*0.017*)**	0.026 (*0.849*)	–
		**[0.052 : 0.484]**	[–0.174 : 0.290]	**[0.066 : 0.546]**	**[0.140 : 0.612]**	[–0.157 : 0.355]	**[0.049 : 0.497]**	[–0.245 : 0.297]	
	MD peak height	–	–	–	–	–	–	–	0.209 (0.*112*)
									[–0.050 : 0.468]
	Model sig. (*P*-value, Adj. R^2^)	**<*0.001*, 0.433**	**<*0.001*, 0.345**	**<*0.001*, 0.299**	**<*0.001*, 0.319**	**0.*001*, 0.202**	**<*0.001*, 0.392**	***0.029*, 0.108**	**0.*004*, 0.162**

All numbers are standardized β-values (*P*-values) (95% standardized β confidence interval (CI)), with overall model significance being given on the bottom row. Models are separated into those which test NAWM and WM metrics horizontally. Significant associations are shown in bold.

The number of lacunes was independently and significantly associated with Global cognition, Processing speed, MoCA and SSQoL in both tissue class models. NBV only maintained a significant association with MoCA and EuroQoL (in both tissue class models). WMH lesion load was no longer significantly related to any outcome measures.

### Systematic review: comparison of strength of associations between DTI and cognition with that from previous studies

The search terms identified 230 papers, and after reading their abstracts, 37 selected for review. An additional five papers were identified from reference lists. Eight of these 42 papers met inclusion criteria [8,12,29–31,38–40]. Supplementary Table S2 details these papers and includes key findings from each study. Of note, one of these [39] is a multicentre study across three sites using identical 1.5T scanners and acquisition sequences, with MoCA and MMSE as cognitive measures.

Two of these papers reported 95% confidence intervals (CI) with their β-values for associations between DTI metrics and cognition [12,38]. Comparing the magnitude of the DTI-based β-values (ignoring direction, as this will be influenced by the specific DTI parameter used, which differs between papers) from the presented study for the same cognitive domain shows that these fell within or were higher than these previously reported CIs for Global cognition (our β =**0.268**, previous CIs = **–0.22** to –**0.06** [12] and **–0.38**–**0.02** [38]) Executive functioning (i.e. Mental flexibility; our β =**0.306**, previous CIs = **–0.16** to **–0.06** [12] and **0.05**–**0.39** [38]), Verbal fluency (our β =**0.376**, previous CIs = **–0.21** to **–0.02** [12]) and Verbal Memory (our β =**0.099**, previous CIs = **–0.28** to **–0.06** [12]). Only the presented β for Processing speed was lower than a previously reported CI (but only in one of these papers; our β =**0.058**, previous CIs = **–0.24** to –**0.06** [12] and **–0.33** to **0.06** [38]). Conversely, previously reported β-values from all the eight papers fell within the CIs found in the presented analyses in all instances except for one case of Verbal memory being greater than our CI (previous β = **–0.86** [31], our CI = –**0.157** to **0.355**) and one case of Verbal fluency being lower than our CI (previous β = **-–.11** [12], our CI = **0.140**–**0.612**).

### Site-specific findings

These analyses are reported in full in the Supplementary material.

In order to assess any variation across individual sites, analyses were conducted on data from each site individually. FA median and MD peak height of each site were compared by one-way ANOVA, which returned a non-significant finding for each (FA: *P*=0.424, MD: *P*=0.148). Comparison of all outcome measures (i.e. cognitive domains, MoCA and QoL scales) between sites by one-way ANOVA and Kruskal–Wallis also showed no significant findings (*P*-value range: 0.192–0.827).

**Figure 2:**
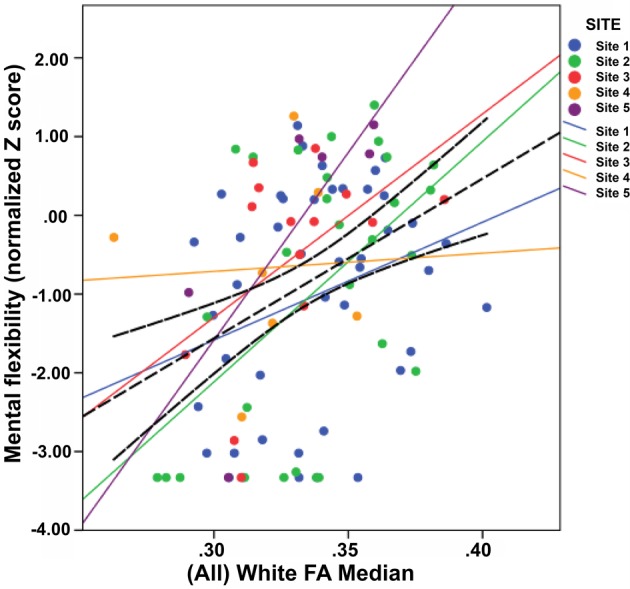
A scatterplot showing the relationship between WM FA median and Mental flexibility, stratified by study site. In addition to individual site regression lines, the regression line for the total is also included with accompanying 95% CI limits (black, dashed line).

‘Complex model’ analyses were also repeated at sites 1, 2 and 3 individually. These were repeated in cases where a DTI metric had been shown to have a significant relationship with a cognitive domain in the main analyses. These relationships were further visualized by scatterplot in *all* sites, with the 95% CI around the total regression line also included for comparison. Complex model results showed sites 1 and 2 to have β-values which were within or higher than the 95% CI limits for the same comparison in the main analyses. While this was also true for site 3 in the Global cognition model, the Mental flexibility and Verbal fluency models gave a lower β-value than the CI limits. The scatterplot with the ‘weakest’ (i.e. flattest) individual site fit is included here as Figure 2. This shows the relationship between WM FA median and Mental flexibility, with a weak fit for site 4 (but not site 3) in that its line falls outside the total CI limits in a manner showing it to be flatter. Supplementary Figures S1 and S2 repeat this scatterplot for Global cognition and Verbal fluency comparisons and likewise indicate site 3 (but not site 4) to have a weak fit in each. All other sites show either good fits (i.e. fall completely within the CI limits; see site 1 in Figure 2) or ‘strong’ ones (i.e. fall outside the total CI limits in a manner showing them to have steeper slopes; see sites 2, 3 and 5 in Figure 2). This suggests that the majority of sites do contribute to the main study findings. It is possible that individual cases of small Complex model β-values, and unusually ‘weak’/‘strong’ scatterplot fits are due to lack of power from low sample sizes.

## Discussion

In this analysis of baseline data from a multicentre clinical trial of SVD, we found associations between DTI metrics and cognition of a similar magnitude to those reported in previous single-centre studies. This provides support for the use of DTI measures as surrogate markers in clinical trials of SVD.

We found that both DTI markers and lacunar infarct count were independently associated with Global cognition and MoCA results. Additionally, DTI markers were independently associated with Mental flexibility and Verbal fluency, and lacunes with Processing speed and SSQoL. In contrast, we found no independent associations between WMH lesion load and cognition and only two for brain volume (with MoCA and EuroQoL). This is in-line with most previous literature from single-centre studies, which have found weak or absent associations between WMH and cognition in patients with severe symptomatic SVD [8,29,39]. However, it has been previously shown, as we also have, that the presence and number of lacunar infarcts [8,38] and the extent of diffuse WM damage assessed on DTI [8,12,29,31,38,39], are the strongest predictors of cognitive functioning. Furthermore, both have been shown to predict risk of dementia in longitudinal studies [41,42], while lacunes and the apparent diffusion coefficient (a diffusion-weighted imaging measure highly similar to MD) have also been shown to predict future cognitive decline [43,44]. Number of lacunes was chosen in the present study instead of lacune volume as it is a more practical measure to obtain in a clinical setting, and similar associations with cognitive performance have been found between these in a comparable severe SVD population [45].

Clinical trials of new agents in SVD will need to be multicentre and if MRI is to be used as a surrogate marker, it is important to evaluate how the different markers perform in a multicentre setting. While research in other neurological disorders such as Parkinson’s [46] and Huntington’s [47] disease have shown that DTI markers of disease can be successfully applied in a mutlicentre study, there have been few studies addressing this issue in SVD. The use of multiple scanners, possibly from different manufacturers, is likely to add noise and may diminish the statistical sensitivity of these metrics.

PRESERVE is one of the first studies to use advanced MRI as a surrogate marker in SVD trials. In this setting, we have shown that the magnitudes of associations between DTI and cognition are highly comparable with previous, single-centre studies, further validating the use of these metrics in this context. Additionally, while WM and NAWM DTI were always significantly associated with the same outcomes, the strengths of these associations was consistently descriptively greater in WM models. This indicates the simpler process of obtaining a WM mask is at least equally valid and may be more practical in a clinical setting. It should also be noted that previous research has indicated through power calculations that DTI parameters could detect change with much smaller sample sizes than lacunes, due to the frequency of new lacunes being relatively low [11]. This suggests that DTI metrics may be the most powerful surrogate marker of the two.

Examination of individual site data did demonstrate some variation in the strength of associations between MR parameters and cognition from different centres, but the majority of these effect sizes were within (or greater than) the expected ranges as determined by 95% CIs for β-values and regression slopes from the main analyses. DTI metrics and cognition did not significantly differ between sites, meaning that it is likely that a lack of power due to a low site sample size was a contributing factor to the instances where this was not the case. The similarity of DTI and cognitive metrics across sites also suggests good comparability between the centres involved in the present study. With respect to the wider literature however, the authors do note that DTI metrics have sometimes been shown to differ in magnitude between manufacturers, such as one paper where MD values were found to be systematically higher on Siemens compared with Philips scanners (this would not affect peak height of MD as used in the presented study, but could affect measures of MD centrality [48]). Another paper [49] examined reproducibility of whole brain MD peak height between a 1.5T and a 3T Siemens scanner in a sample size of seven CADASIL patients, which achieved an intraclass correlation coefficient of 0.752 (indicating ‘good’ reliability [50]). A further paper has found that scanner upgrades to affect DTI after scanning CADASIL patients [51]. These findings show that caution should be used when combining DTI data from different manufacturers or when taking measurements over time and future research may wish to take this into account in analyses. These considerations also highlight the importance of conducting multicentre scanner calibration and standardization of acquisition protocols prior to study commencement, as well as on-going quality control checks during the study duration in multicentre research of this nature.

There were some limitations to the present study. There were variable sample sizes across sites, meaning the influence of some centres is much stronger than others on our findings. In particular, having a greater number of participants scanned on non-Philips hardware would have provided more information about the comparability across scanners. The lack of data on inter-scanner reproducibility is also limiting and would have been valuable in more closely judging the sensitivity of these metrics across sites. It would also have been advantageous to acquire a field map with the DTI protocol so that corrections for susceptibility induced distortions could have been made. However registration to DTI space did appear good, so this is unlikely to have caused any major problems.

To conclude, in a multicentre study, we have shown that DTI metrics and lacune count correlate with cognition to a similar degree to that found in single-centre studies. Our findings support the use of DTI as a surrogate marker of SVD in multicentre studies.

## Clinical perspectives

- DTI metrics have been suggested as clinical trial markers of SVD, but studies investigating this have been single-centre and as clinical trials are likely to be multicentre. We investigate the associations between these metrics and cognition in a multicentre clinical trial of SVD.
- In a cross-sectional analysis, significant associations were found between white matter DTI metrics and cognition; these were of a comparable magnitude with those previously reported in single-centre studies.
- These findings support the use of DTI metrics as markers of SVD in multicentre clinical trials.

## Abbreviations

BET: brain extraction tool
CABG: coronary artery bypass graft
CI: confidence interval
CSF: cerebrospinal fluid
DSC: digit symbol coding
DTI: diffusion tensor imaging
FA: fractional anisotropy
FLAIR: fluid-attenuated inversion recovery
FSL: FMRIB software library
GM: grey matter
MD: mean diffusivity
MoCA: Montreal cognitive assessment
NART: National Adult Reading Test
NAWM: normal appearing white matter
NBV: normalised brain volume
QoL: quality of life
RAVLT: Rey auditory verbal learning test
SSQoL: stroke specific quality of life
SVD: small vessel disease
TMT: trail making test
TPM: tissue probability map
T1W: T1-weighted
T2*W: T2*-weighted
WM: (all) white matter
WMH: white matter hyperintensity
